# Natural infection versus hybrid (natural and vaccination) humoral immune response to SARS-CoV-2: a comparative paired analysis

**DOI:** 10.3389/fimmu.2023.1230974

**Published:** 2023-09-01

**Authors:** Laila AbdelWareth, Farida Alhousani, Rowan Abuyadek, James Donnelly, Andrea Leinberger-Jabari, Shereen Atef, Rami H. Al-Rifai

**Affiliations:** ^1^ National Reference Laboratory, Abu Dhabi, United Arab Emirates; ^2^ Pathology & Laboratory Medicine Institute (PLMI), Cleveland Clinic Abu Dhabi, Abu Dhabi, United Arab Emirates; ^3^ Abu Dhabi Public Health Center (ADPHC), Abu Dhabi, United Arab Emirates; ^4^ High Institute of Public Health, Alexandria University, Alexandria, Egypt; ^5^ Public Health Research Center, New York University, Abu Dhabi, United Arab Emirates; ^6^ Faculty of Medicine, Ain Shams University, Cairo, Egypt; ^7^ Institute of Public Health, College of Medicine and Health Sciences, United Arab Emirates University, Al Ain, United Arab Emirates

**Keywords:** SARS-CoV-2, vaccination, spike antibodies, neutralizing antibodies, hybrid immunity

## Abstract

**Objectives:**

There is substantial immunological evidence that vaccination following natural infection increases protection. We compare the humoral immune response developed in initially seropositive individuals (naturally infected) to humoral hybrid immune response (developed after infection and vaccination) in the same population group after one year.

**Methods:**

The study included 197 male individuals who were naturally infected with SARS-CoV-2 and then vaccinated with SARS-CoV-2 vaccine. Trimeric spike, nucleocapsid, and ACE2-RBD blocking antibodies for SARS-CoV-2 were measured. Nasal swabs were collected for SARS-CoV-2 PCR testing. Information on vaccination against SARS-CoV-2 and PCR verified infection was retrieved from official databases (Abu Dhabi Health Data Services- SP LLC. (“Malaffi”), including number of vaccine doses received, date of vaccination, and type of the received vaccine.

**Results:**

All the study population were tested PCR-Negative at the time of sample collection. Our results showed that there was a significant rise in the mean (SD) and median (IQR) titers of trimeric spike, nucleocapsid and ACE2-RBD blocking antibodies in the post-vaccination stage. The mean (± SD) and median (IQR) concentration of the anti-S antibody rose by 3.3-fold (+230% ± 197% SD) and 2.8-fold (+185%, 220–390%, p<0.001), respectively. There was an observed positive dose-response relationship between number of the received vaccine doses and having higher proportion of study participants with higher than median concentration in the difference between the measured anti-S and ACE2-RBD blocking antibodies in the post-vaccination compared to pre-vaccination.

**Conclusion:**

Our study demonstrates that COVID-19 vaccination post natural infection elicits a robust immunological response with an impressive rise of SARS-CoV-2 antibodies, especially the ACE2-RBD blocking antibodies.

## Highlights

• More than 2-fold significant increase in all immunoglobulin titers after vaccination.
• All immunoglobulin concentrations (titers) where higher in the first 60 days after vaccinations in participants with hybrid immunity.
• Previous SARS-CoV-2 infection alone doesn’t elicit sufficiently protective immune response.

## 1 Introduction

The development of various vaccines against SARS-CoV-2 virus has provided a significant protection measure against the virus. Most studies published on the effectiveness of these COVID-19 vaccines have shown that vaccination reduced hospitalizations and development of complications in infected individuals (1–3). Some studies discussed the potential for adverse effects of the vaccines especially during the early stages of vaccines introduction. After administration of billions of doses of these vaccines all over the world, almost all studies suggested that there are no significant side effects with the administration of these vaccines rather than the regular effect that might occur with any other vaccines (4).

Centers for Disease Control and Prevention (CDC) recommends COVID-19 vaccination for all eligible persons, including those who have been previously infected with SARS-CoV-2 (5). Some experts estimate that vaccinating 70% to 85% of the population worldwide could enable a return to normalcy. We are currently far from this goal. Still there are many COVID-19 vaccine hesitant individuals due to misinformation, or other reasons (6). As well, access to vaccines remains an issue in some countries. Those group are really very important because they allow for viral mutation, and this facilitates the development of new variants. In fact, these new variants negatively impact the efficacy of the vaccines currently available and require the periodic updating of the vaccine.

The United Arab Emirates (UAE), which hosts the world’s most fully vaccinated population (7), has given five types of vaccines since the emergency use approval to control the spread of the SARS-CoV-2 virus. BBIBP-CorV (commercial name: Covilo, Sinopharm’s Beijing Institute of Biological Products), was the first vaccine got approved at UAE, followed by others as BNT162b2 (commercial name: Comirnaty, Pfizer-BioNTech), rAd26-S + rAd5-S (commercial name: Sputnik V, Gamaleya Research Institute of Epidemiology and Microbiology), ChAdOx1-S (commercial name: Vaxzevria, AstraZenecaUniversity of Oxford), and mRNA-1273 (commercial name: Spikevax, Moderna-NIAID) (8). Knowledge on the duration of vaccine-induced antibody responses by different vaccines types and their efficacy is essential for making rational decisions regarding immunization strategies and booster doses administration especially for high risk population.

In our study we aimed to compare the humoral immune response developed in initially seropositive individuals (naturally infected) to humoral hybrid immune response (developed after infection and vaccination) in the same population group after one year.

## 2 Materials and methods

### 2.1 Study design and samples collection

#### 2.1.1 Enrolment, laboratory samples and survey data collection

All our participants (197 individuals) were initially seropositive (positive to anti-S and anti-N Abs) for SARS-CoV-2 in an initial seroprevalence study conducted by Alsuwaidi and his colleagues (9). At enrolment, an online self-administered survey questionnaire was employed using the Voxco™ survey software customized to our specifications. All of the consenting participants filled out an interview questionnaire and consented to the collection of whole blood sampling and a nasopharyngeal swab for SARS-CoV-2 PCR testing. Collected samples were preserved according to manufacture recommendation. Information on vaccination against SARS-CoV-2 and PCR verified infection was retrieved from official databases (Abu Dhabi Health Data Services- SP LLC. (“Malaffi”), 2022 (10), including number of vaccine doses received, date of vaccination, and type of the received vaccine.

### 2.2 Laboratory Testing

#### 2.2.1 SARS-CoV-2 and ACE 2 receptor binding domain region (blocking) antibody immunoassays

The DiaSorin LIAISON^®^ SARS-CoV-2 Trimeric S immunochemiluminescent assay was performed on blood sera using the LIAISON^®^ XL analyzer (DiaSorin S.p.A, Saluggia, Italy).The DiaSorin assay is traceable to the WHO first International Standard for SARS-CoV-2 antibody quantitation and is reported in Binding Antibody Units (BAU/mL). The SARS-CoV-2 nucleocapsid total antibodies were analysed on the Roche Cobas 6000 platform (Roche Diagnostics International AG, Rotkreuz, Switzerland. For measuring the antibodies directed against the ACE2 SARS-CoV-2 Receptor Binding Domain (RBD), we used iFlash-2019-nCoV surrogate ‘neutralizing’ antibody kit, a one-step competitive chemiluminescence immunoassay on the iFlash 1800 analyzer (YHLO Biotech Co., Ltd., Shenzhen, China). This assay is configured to measure the decrease in the binding of ACE2 to the ACE2 receptor binding domain (RBD) which indicates the presence of antibodies, potentially of any type (G, A or M) that attenuate ACE2 – receptor binding to a recombinant RBD protein. All the assays were performed according to the manufacturer’s instructions.

Any antibody results exceeding the analytical measuring range were not repeated diluted to find the final titer. This final titer was not required for our purposes of determining the shifts in values post convalescence and vaccination.

#### 2.2.2 SARS-CoV-2 PCR

Viral RNA was extracted and detected using the NeoPlex™ Covid-19 Detection Kit (RT)-PCR detecting the target genes N gene and ORF1a (SolGent Co., Ltd. Daejeon, Korea).

#### 2.2.3 Statistical analyses

Frequency distributions and proportions of the categorical measured characteristics while mean and standard deviation (SD) of the continuous characteristics were described. The post-natural infection and before vaccination (hereafter referred as ‘pre-vaccination’) as well as the post-natural infection and post-vaccination (hereafter referred as ‘post-vaccination’) distribution of the measured three humoral immune biomarkers (anti-S, anti-N, and ACE2 blocking antibodies) was described. The pre-vaccination and post-vaccination concentration of the measured three humoral biomarkers was described using mean ± standard deviation (SD) and median and interquartile range (IQR). The later distribution plotted in Boxplots.

Normality assumption for the distribution values of the antibodies was investigated using the Kolmogorov-Smirnov test. Difference between the post-vaccination and pre-vaccination mean and median concentrations of the three measured antibodies was evaluated. To assess the difference in the mean concentrations of the antibodies, the p-value was extracted from the paired-samples *t*-test of two related samples. To assess the difference in median titers of the antibodies, the P-value was obtained from Wilcoxon Signed Ranks test assessing difference in distribution of non-normality distributed and two-related samples.

In the pre-vaccination, post vaccination, and between pre-vaccination and post-vaccination, difference in the median concentration of the antibodies, by time since last vaccine dose received, was evaluated and p-value extracted from Kruskal-Wallis test assessing the difference between groups of non-normally distributed data and Wilcoxon Signed Ranks test assessing difference in distribution of non-normality distributed and two-related samples. Same analysis was repeated after excluding individuals with repeated infection with SARS-CoV-2 before receiving the first vaccine dose.

The strength of correlation between the same antibody type in the pre-vaccination and post-vaccination and between the three measured antibodies in the pre-vaccination and post-vaccination was evaluated by Spearman correlation test. Correlation between the different titers in the measured antibody concentrations in post-vaccination compared to pre-vaccination was also evaluated using Spearman correlation test. These explored correlations are plotted in matrix scatter plots.

To explore factors contributing to producing equal or more than the median concentration in the difference between post-vaccination and pre-vaccination titer, the quantified difference in concentrations was categorized into a binary outcome (< median change and ≥ median change). Correlation between the measured characteristics including history of vaccination and the binary outcome for each antibody type (N, S, ACE2 – RBD blocking) concentration was investigated. Chi-squared or Fisher’s exact tests were used for categorical characteristics, and the two-sample non-parametric Mann-Whitney U test was used for continuous characteristics.

SPSS IBM Statistics (v26) software was used. P-values <0.05 were considered statistically significant.

#### 2.2.4 Ethical considerations

The study was approved by the UAE National COVID-19 Research Ethics Committee (reference number: DOH/CVDC/2021/856 and amendment number: DOH/CVDC/2021/1703). From each participant, consent to collect survey information, blood sample, and nasopharyngeal swab, was obtained.

## 3 Results

### 3.1 Study population

The study included 197 male individuals who were naturally infected with SARS-CoV-2 and then vaccinated with SARS-CoV-2 vaccine. All the study population were tested PCR- Negative at the time of sample collection. The study participants had a mean age of 34.50 ± 8.3 years, majority (99.5%) were Asians, 56.3% had primary schooling or below, 29.4% were current or ex-smokers, and with a mean BMI of 24.6 ± 3.7 kg/m^2^. Having at least one chronic comorbidity was reported by 11.2% of the participants. Twenty (10.2%) participants were re-infected with SARS-CoV-2 in the past 12 months prior to receiving the first vaccine dose. Two-thirds (68.0%) of the study participants received three vaccine doses, 30.5% received only two vaccine doses, and only three participants (1.5%) received only a vaccine dose. The majority (92.4%) of the participants were vaccinated with BBIBP-CorV (Sinopharm) vaccine type, 11 (5.6%) received rAd26-S+rAd5-S (Sputnik V Gam-COVID-Vac), and four (2.0%) received mixed vaccine types. The mean time duration since the last received vaccine dose and post-vaccination measurement was 109.5 (± 63.2 SD) days (**Table 1**).

**Table 1:** Distribution of the study population by their measured sociodemographic, clinical, SARS-CoV-2 vaccination characteristics by lower than the median or equal/higher than the median level in difference between the hybrid and natural immune response.

	Total (N =197) n (valid %)	Difference in the hybrid relative to the natural immune response								
		Anti-S BAU/ml (n= 195)		*P*-value	Anti-N COI (n= 154)		*P*-value	ACE2 blocking Ab AU/ml (n= 197)		*P*-value
		< Median change (n = 97, valid %)	≥ Median change (n=98, valid %)		< Median change (n = 75, valid %)	≥ Median change (n = 79, valid %)		< Median change (n = 98, valid %)	≥ Median change (n = 99, valid %)	
Age median, IQR – year (range, mean ± SD)	34.0, 27.0–41.0 (20–55, 34.50 ± 8.3) years	34.5 ± 8.4	34.4 ± 8.3	0.838^a^	36.01 ± 8.6	34.2 ± 8.4	0.270^a^	35.07 ± 8.5	33.8 ± 8.2	0.300^a^
Nationality				0.380^b^			0.513^b^			0.497^b^
Asian	196 (99.5)	96 (37.6)	98 (62.4)		75 (49.0)	78 (51.0)		97 (49.5)	99 (50.5)	
African	1 (0.5)	1 (100.0)	0 (0.0)		0 (0.0)	1 (100.0)		1 (100.0)	0 (0.0)	
Education				0.387^c^			0.387^c^			0.499^c^
Primary schooling and below	111 (56.3)	53 (48.6)	56 (51.4)		47 (50.0)	47 (50.0)		56 (50.5)	55 (49.5)	
Secondary schooling	74 (37.6)	40 (54.1)	34 (45.9)		26 (50.0)	26 (50.0)		38 (51.4)	36 (48.6)	
University and postgraduate level	12 (6.1)	4 (33.3)	8 (66.7)		2 (25.0)	6 (75.0)		4 (33.3)	8 (66.7)	
Tobacco smoking				0.324^c^			0.070^c^			0.055^c^
Current (48) or ex-smoker (10)	58 (29.4)	32 (55.2)	26 (44.8)		15 (36.6)	26 (63.4)		35 (60.3)	23 (39.7)	
Never smoke	139 (70.6)	65 (47.4)	72 (52.6)		60 (53.1)	53 (46.9)		63 (45.3)	76 (54.7)	
Received flu shot										
Yes	1 (0.5)	–	–		–	–		–	–	
No	196 (99.5)	–	–		–	–		–	–	
BMI, median, IQR (mean ± SD) kg/m	24.3, 21.9–27.0 (24.6 ± 3.7)			0.465^c^			0.125^c^			0.123^c^
Underweight	9 (4.9)	3 (33.3)	6 (66.7)		1 (14.3)	6 (85.7)		2 (22.2)	7 (77.8)	
Normal weight	100 (54.1)	48 (48.5)	51 (51.5)		37 (47.4)	41 (52.6)		49 (49.0)	51 (51.0)	
Overweight	58 (31.4)	29 (50.0)	29 (50.0)		25 (56.8)	19 (43.2)		30 (51.7)	28 (48.3)	
Obese	18 (9.7)	11 (64.7)	6 (35.3)		9 (64.3)	5 (35.7)		11 (61.1)	7 (38.9)	
* Missing*	12									
Chronic comorbidities				0.042^c^			0.249^c^			0.688^c^
No	174 (88.8)	90 (52.0)	83 (48.0)		63 (46.7)	72 (53.3)		87 (50.0)	87 (50.0)	
Yes, at least one	22 (11.2)	6 (28.6)	15 (71.4)		11 (61.1)	7 (38.9)		10 (45.5)	12 (54.5)	
* Missing*	1									
Re-infection with SARS-CoV-2				0.019^c^			0.701			0.164^c^
No	177 (89.8)	92 (52.6)	83 (47.4)		67 (49.3)	69 (50.7)		91 (51.4)	86 (48.6)	
Yes	20 (10.2)	5 (25.0)	15 (75.0)		8 (44.4)	10 (55.6)		7 (35.0)	13 (65.0)	
Vaccination against SARS-CoV-2				0.732^c^			0.818^c^			0.392^c^
Only one dose	3 (1.5)	2 (66.7)	1 (33.3)		1 (33.3)	2 (66.7)		1 (33.3)	2 (66.7)	
Two doses	60 (30.5)	28 (46.7)	32 (53.3)		22 (51.2)	21 (48.8)		26 (43.3)	34 (56.7)	
One booster dose – three doses	134 (68.0)	67 (50.8)	65 (49.2)		52 (48.1)	56 (51.9)		71 (53.0)	63 (47.0)	
Boosted vs not-boosted (n = 194)^1^				0.356^b^			0.738^c^			0.214^c^
Not boosted - two doses only	60 (30.5)	28 (46.7)	32 (53.3)		22 (51.2)	21 (48.8)		26 (43.3)	34 (56.7)	
Boosted with only one dose	134 (69.1)	67 (50.8)	65 (49.2)		52 (48.1)	56 (51.9)		71 (53.0)	63 (47.0)	
Vaccine type				<0.001			0.156			0.026
BBIBP-CorV (Sinopharm)	182 (92.4)	97 (53.9)	83 (46.1)		71 (47.7)	78 (52.3)		96 (52.7)	86 (47.3)	
One dose	3 (1.6)	2 (2.1)	1 (1.2)	0.578	1 (1.4)	2 (2.6)	0.876	2 (2.1)	1 (1.2)	0.578
Two doses	47 (25.8)	28 (28.9)	19 (22.9)		19 (26.8)	20 (25.6)		28 (28.9)	19 (22.9)	
Three doses	132 (72.5)	67 (69.1)	63 (75.9)		51 (71.8)	56 (71.8)		67 (69.1)	63 (75.9)	
rAd26-S+rAd5-S (Sputnik)	11 (5.6)	0 (0.0)	11 (100.0)		3 (100)	0 (0.0)		1 (9.1)	10 (90.9)	
Mixed vaccine types	4 (2.0)	0 (0.0)	4 (100.0)		1 (100.0)	1 (100.0)		1 (33.3)	3 (66.7)	
Duration since last vaccine dose and post-vaccination antibody testing, median (IQR), range, (mean ± SD)	95.0 (72.0–130.5), 4.0–295 (109.5 ± 63.2)			0.514			0.155			0.678
1 – 14 days	5 (2.5)	3 (60.0)	2 (40.0)		4 (100)	0 (0.0)		2 (40.0)	3 (60.0)	
15 – 30 days	8 (4.1)	4 (50.0)	4 (50.0)		2 (33.3)	4 (66.7)		5 (62.5)	3 (37.5)	
31 – 60 days	20 (10.2)	6 (33.3)	12 (66.7)		6 (40.0)	9 (60.0)		8 (40.0)	12 (60.0)	
61 – 295 days	164 (83.2)	84 (51.2)	80 (48.8)		63 (48.8)	66 (51.2)		83 (50.6)	81 (49.4)	

^1^for those who have received two doses as they are eligible to be boosted.

a P value extracted from Non-parametric Mann-Whitney U test comparing distribution across groups.^b^ P value extracted from the Fisher’s exact test.^c^ P value extracted from the Chi-square test.

### 3.2 Pre- and post-vaccination anti-S, anti-N, and ACE2-RBD blocking antibodies concentration

**Figure 1:**
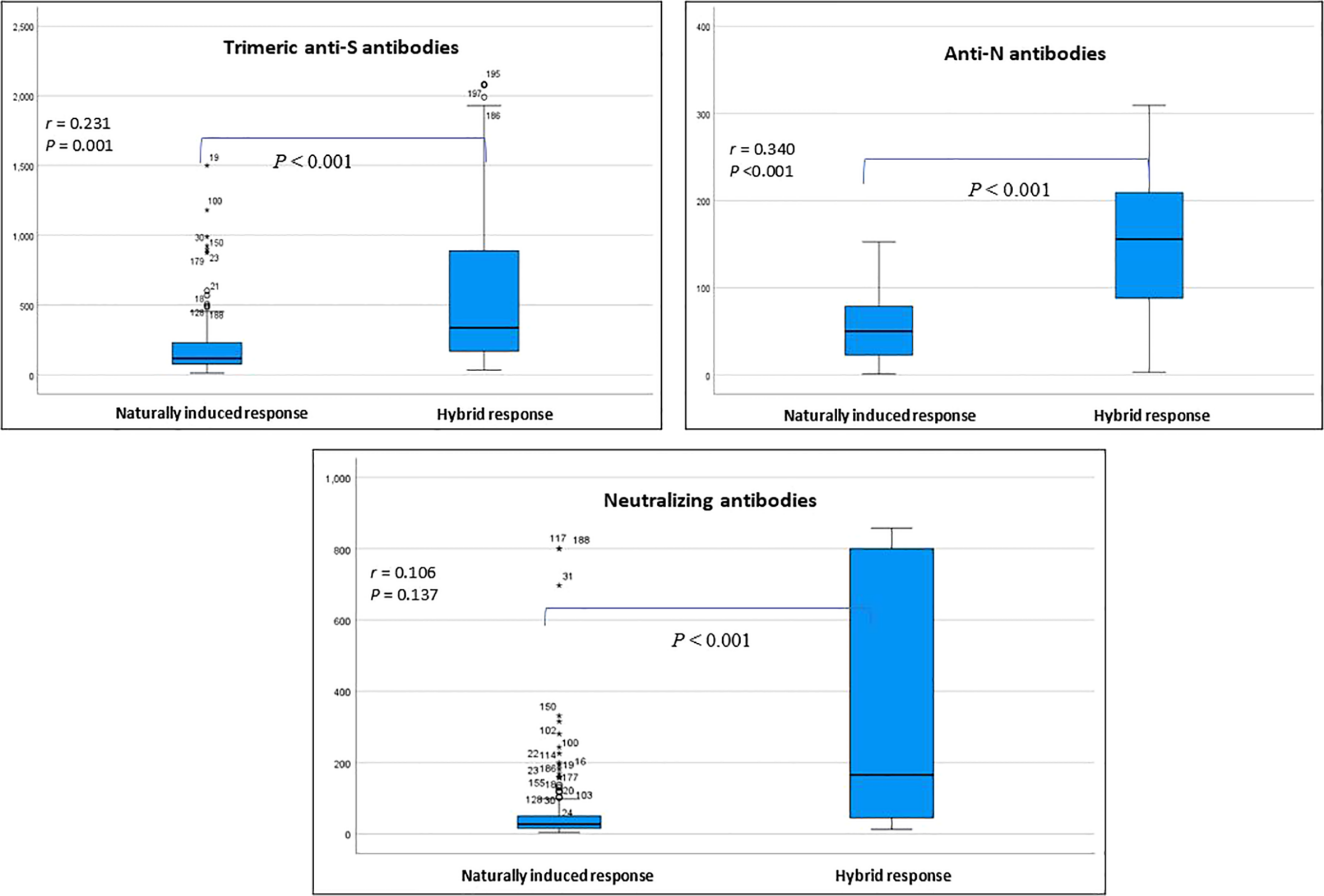
Distribution of the measured three classes of antibodies in the pre-vaccination and post-vaccination stage. Correlation coefficient (*r*) assesses the strength of correlation between same antibody class in the two stages (Spearman correlation test). * outlier.

**Figure 2:**
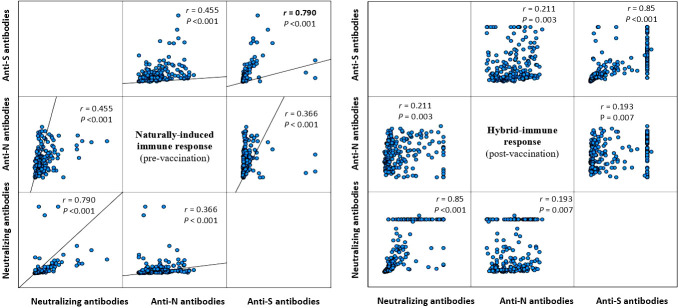
Correlation between the measured three antibody classes during the natural infection (left figure) and the post-vaccination (right figure) stage (Spearman correlation).

**Figure 3:**
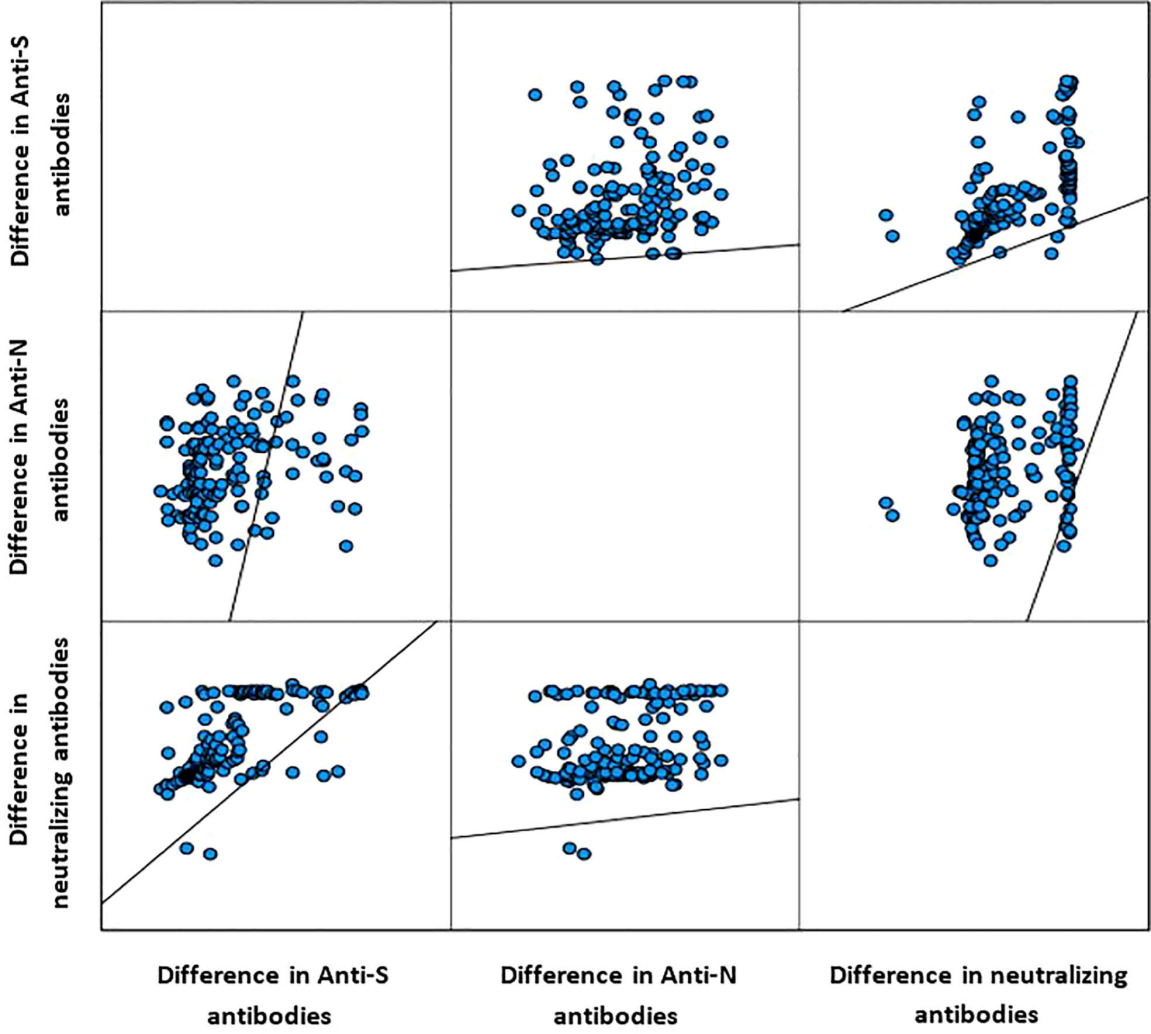
Correlation between the difference in level of the measured three antibody classes (Spearman correlation).

**Table 2** presents the change in the concentrations of the three measured antibodies in post-vaccination period compared to pre-vaccination. There was a significant rise in the mean (SD) and median (IQR) concentration of the three measured antibodies in the study period post-vaccination. The mean (± SD) and median (IQR) concentration of the anti-S antibody rose by 3.3-fold (+230% ± 197% SD) and 2.8-fold (+185%, 220–390%, p<0.001), respectively. The mean (± SD) and median (IQR) concentration of the anti-N antibody rose by 2.6-fold (+161% ± 1.1%, p<0.001) and 2.9-fold (+190%, 44.2–145.2%, p<0.001), respectively. The mean (± SD) and median (IQR) concentration of the ACE2-RBD blocking antibodies increased by more than 6-fold (+500% ± 220%, p<0.001) and (+515, 196–1,490%, p<0.001), respectively. Graphically, the distribution of the measured three antibodies in the convalescent, pre-vaccination and post-vaccination periods are presented in **Figure 1**. Anti-S (r =0.23, p<0.001) and anti-N (r =0.34, p<0.001) concentration in the pre-vaccination were weakly positively correlated with anti-S and anti-N concentration in the post vaccination stage. ACE2-receptor blocking antibody concentrations in pre-vaccination individuals was not correlated (r = 0.11, p=0.137) with that in the post-vaccination period (**Figure 1**). In both pre-vaccination and post-vaccination, the anti-S and ACE2-RBD blocking antibody concentrations were strongly positively (r = 0.79 and 0.85, p<0.001, respectively) correlated (**Figure 2**. The positive correlation between the difference in levels of these two antibodies classes during the natural infection period and the hybrid immune response period was also significant (r = 0.75, p<0.001) (**Figure 3**).

**Table 2:** Distribution of the three measured antibodies (anti-S, anti-N, & ACE2 receptor blocking Abs) in post-natural infection (pre-vaccination) and hybrid immune response (post-vaccination) to SARS-CoV-2 and the difference in the level between the two immune responses.

	Anti-S - BAU/ml			P-value	Anti-N - COI			P-value	Nabs - AU/ml			P-value
	Natural immune response (n = 195)	Hybrid-immune response (n = 197)	Difference (n = 195) [% of change]		Natural immune response (n = 154)	Hybrid-immune response (n = 197)	Difference (n = 154) [% of change]		Natural immune response (n = 197)	Hybrid-immune response (n = 197)	Difference (n = 197) [% of change]	
Mean ± SD	189.2 ± 204.9	624.5 ± 609.1	434.5 ± 600.6 [230 ± 197]	0.003^a^	54.9 ± 38.4	143.72 ± 80.2	97.0 ± 74.2 [161 ± 1.1]	<0.001^a^	56.2 ± 103.6	338.8 ± 331.5	282.6 ± 343.8 [500 ± 220]	<0.001^a^
Median (IQ)	118.0 (75.9–231.0)	336.0 (169.5–912.5)	181.9 (27.0–668.0) [185, 220–390]	<0.001^b^	50.0 (22.6–79.1)	145.0 (81.5–207.0)	90.7 (44.2–145.2) [190, 44.2–145.2]	<0.001^b^	26.8 (15.2–50.3)	165.0 (45.0–800.0)	125.5 (12.7–735.2) [515, 196–1490]	<0.001^b^
Range	13.5–1,500.0	35.0–2,080.0	-416–2054.4		1.2–152.5	2.0–309	-74.2–257.1		4.0–800.0	12.0–858.0	-723.0–843.9	

Re-infection with SARS-CoV-2												
	Median (IQ)	Median (IQ)	Difference (IQR) [% of change]	P-value^b^	Median (IQ)	Median (IQ)	Difference (IQR) [% of change]	P-value^b^	Median (IQ)	Median (IQ)	Difference (IQR) [% of change]	P-value^b^
Yes	178.5 (70.0–273.0)	845.0 (333.0–1,262)	548.5 (182.6–1,189) [370.3%]	<0.001	27.4 (9.8–72.7)	172.5 (68.8–223.3)	106.1 (47.5–208.5) [529.5%]	<0.001	32.4 (13.3–73.7)	694.0 (120.5–800.0)	517.1 (64.6–766.7) [2,041%]	<0.001
No	114.0 (75.9–230.0)	324.0 (161.5–840.0)	160.14 (16.4–585.0) [184.2%]	<0.001	51.3 (24.1–80.5)	142.0 (81.5–205.0)	89.7 (43.1–144.8) [176.8%]	<0.001	25.5 (15.5–48.6)	139.0 (42.0–800.0)	115.5 (11.3–781.01) [445.1%]	<0.001
P-value^c^	0.417	0.008	0.007		0.75	0.437	0.216		0.531	0.008	0.090	

Duration since the last received vaccine dose and post-vaccination antibody testing												
	Median (IQ)	Median (IQ)	Difference (IQR) [% of change]	P-value^b^	Median (IQ)	Median (IQ)	Difference (IQR) [% of change]	P-value^b^	Median (IQ)	Median (IQ)	Difference (IQR) [% of change]	P-value^b^
1 – 60 days	110.0 (66.7–319.0)	457.0 (167.0–784.5)	(n = 31) 214.0 (28.5–553.3) [315.5%]	0.002	66.4 (22.7–96.5)	158.0 (68.0–204.0)	(n = 25) 80.5 (34.3–140.3) [138.0%]	<0.001	21.8 (14.6–79.3)	173.0 (49.5–800)	(n = 31) 142.9 (30.0–785.7) [693.6%]	<0.001
61 – 295 days	119.5 (78.7–219.5)	327.0 (169.0–935.0)	(n = 164) 180.4 (24.3–701.0) [173.6%]	<0.001	49.1 (22.2–74.0)	153.0 (90.0–215.0)	(n = 129) 90.7 (45.7–145.6) [211.0%]	<0.001	27.0 (15.5–49.6)	165.0 (45.0–800.0)	(n = 164) 119.7 (10.1–732.8) [511.1%]	<0.001
P-value^c^	0.978	0.510	0.978		0.366	0.475	1.00		0.855	0.855	0.432	

Normality assumption testing: Kolmogorov-Smirnov test (n > 50).

Anti-S: pre-vaccination (p<0.001), post-vaccination (p<0.001), and difference (p<0.001) are non-normally distributed.

Anti N: pre-vaccination (p<0.002) and post-vaccination (p=0.023) are non-normally distributed while difference was normally distributed (p=0.200).

ACE2 blocking Abs pre (p<0.001) and post-vaccination (p<0.001), and difference (p<0.001) are non-normally distributed.

a P-value obtained from Paired-samples T test assessing difference in means of two-related samples.^b^ P-value obtained from Wilcoxon Signed Ranks test assessing difference in distribution of non-normality distributed and two-related samples.^c^ Kruskal-Wallis test assessing difference between groups of non-normally distributed data. IQR: interquartile range.

Irrespective of the timing when the last vaccine dose was received relative to the timing of measuring the titers in the post-vaccination stage, all study participants had the same titer of the three types of SARS-CoV-2 antibodies before vaccination. The magnitude of increase in the three measured antibody titers was also similar (p= 0.978) between individuals who had received the last vaccine dose 1-60 days or > 60 days before the measuring the in the post-vaccination stage. In pre-vaccination stage, there was no significant difference in titer of the three measured by re-infection status in the past 12 months prior to receiving the first vaccine dose. In the post-vaccination stage, study participants with PCR-confirmed re-infection with SARS-CoV-2 had significantly higher anti-S (370.3% vs 184.2% increase, respectively) and blocking (2,041% vs 445.1% increase, respectively) antibodies (**Table 2**).

There was an observed positive dose-response relationship between number of the received BBIBPCorV (Sinopharm) vaccine doses and having higher proportion of study participants with higher than median concentration in the difference between the measured anti-S and ACE2-RBD blocking antibodies in the post-vaccination compared to pre-vaccination. All the participants who had received only rAd26-S+rAd5-S (Sputnik) vaccine had higher than the median titer increase in the anti-S and ACE2-RBD blocking antibodies, respectively, and all of them were never re-infected with SARS-CoV-2 prior to vaccination (**Table 1**).

## 4 Discussion

The relationship between protection against SARS-CoV-2 infection and the titer of ACE2-RBD blocking antibodies has been demonstrated by several studies (11–13) all agreed that the blocking antibody titer is highly predictive of immune protection.Our data showed that there was a significant rise in the titer of the three measured antibodies, yet the most significant increase was encountered with the titer of ACE2 receptor - RBD blocking antibodies which showed more than 6 fold increase after vaccination group. In addition to that we observed also that the titer of ACE2 blocking antibodies has a positive vaccine dose response relationship.

Our study is unique in that the workers residing in company sponsored accommodations served as an accelerated model for SARS-CoV-2 transmission and re-transmission in the general population. Here we show that the titers of three SARS-CoV-2 were significantly increased in the study population who were re-infected with SARS-CoV-2 before receiving the vaccine than those who didn’t become re-infected again before vaccination.

In this study, we reported that two vaccine doses elicit high titers of SARS-CoV-2 anti-S antibody and ACE2-RBD blocking antibodies titers. Importantly, the third vaccine dose significantly increases the three antibody titers especially the ACE2-RBD blocking antibodies. We have previously reported that multiple doses of COVID-19 vaccines are likely to increase the number and quality of antibody production and memory B cells should be more efficacious in preventing reinfection when compared with a single dose of vaccine irrespective of most variant changes (14). We have also demonstrated that an increase in the number of vaccine doses by one dose was associated with increased odds of having more than the median concentration of the antibodies (14).

Regarding the association between the type of the SARS-CoV-2 vaccine and the immune status, several studies showed that the concentrations of the measured antibodies varied according to the different types of studied vaccines, (even after controlling for potential confounders as number of vaccine doses and number of days after vaccination) (14, 15). The majority of our cohort received the Sinopharm vaccine as it was the first to get approval for emergency use in UAE, all of the study participants who had received only rAd26-S+rAd5-S (Sputnik) vaccine had higher than the median titer increase in the anti-S and ACE2-RBD blocking antibodies, respectively, and all of them were never re-infected with SARS-CoV-2 during study period. In fact, in the initial stages of the pandemic when no vaccines were available, there is no doubt that the emergency authorization and use of available non-mRNA-based vaccines played a significant role in alleviating the burden of the pandemic.

In the post-vaccination stage, study participants with PCR-confirmed re-infection with SARS-CoV-2 had significantly higher anti-S and ACE2-RBD blocking antibodies. This is definitely emphasizing on the role of memory B cell response in developing of immunity, similar finding had been reported by Timothy and his colleagues who found that vaccination after recovery from natural SARS-CoV-2 infection, or “hybrid immunity,” has been reported to substantially increase both the potency and breadth of humoral response to SARS-CoV-2 (16).

Limitations of our work includes that by design we chose not to measure antibody production to the final endpoint through repeated dilutions. Many of the vaccinated post convalescent individuals exceeded the analytical measuring range. Our interest was in determining the shift in the values in the cohort and not the absolute values. The *r* value of the correlations are to some extent impacted by this, however, the significance of the shift is not. Also, at the time of collection of the initial convalescent time point there wasn’t a T Cell assay to specific to SARS-CoV-2 and therefore we couldn’t assess cellular response. All of the vaccines are based on the initial Wuhan variant and subsequent variants that successfully gained prominence inherently had variations in their antigens which would likely impact antibody recognition to the virus. Lastly, we used a male cohort as the guest workers in the housing was limited to males.

## Conclusion

Our study demonstrates that COVID-19 vaccination post natural infection elicits a robust immunological response particularly with an impressive rise of SARS-CoV2 antibodies, especially the ACE2-RBD blocking antibodies. The type of vaccine used will have an impact on re-infection rates however, all vaccines greatly increased the titers of ACE2-RBD blocking antibodies.

## Data availability statement

Data would be available from the corresponding authors based on justifiable request and after approval of the ethical committee and the Abu Dhabi Public Health Center.

## Ethics statement

The study was approved by the UAE National COVID-19 Research Ethics Committee (reference number: DOH/CVDC/2021/856 and amendment number: DOH/CVDC/2021/1703). From each participant, written informed consent to collect survey information, blood sample, and nasopharyngeal swab, was obtained.

## Author contributions

The study designed and conceived by LA, SA, and JD. SA, and JD performed laboratory tests and provided the data. All authors contributed to questionnaire development, data collection, extracted the data from medical records, and coded the data. RA-R analyzed the data and interpreted results. All authors contributed to the study design and procedure, and interpreting findings. RA-R, SA, and JD wrote the manuscript, and all coauthors provided input. All authors read and approved the final manuscript.
